# Rheology and Tack Properties of Biodegradable Isodimorphic Poly(butylene succinate)-*Ran*-Poly(ε-caprolactone) Random Copolyesters and Their Potential Use as Adhesives

**DOI:** 10.3390/polym14030623

**Published:** 2022-02-06

**Authors:** Aleida J. Sandoval, María Mercedes Fernández, María Virginia Candal, Maryam Safari, Antxon Santamaria, Alejandro J. Müller

**Affiliations:** 1 Laboratorio de Procesamiento de Alimentos, Departamento de Tecnología de Procesos Biológicos y Bioquímicos, Universidad Simón Bolívar, Caracas 1080-A, Venezuela; 2 POLYMAT and Department of Polymers and Advanced Materials: Physics, Chemistry and Technology, Faculty of Chemistry, University of the Basque Country, 20018 Donostia-San Sebastián, Spain; mercedes.fernandez@ehu.eus (M.M.F.); mariavirginia.candalp@ehu.eus (M.V.C.); maryam.safari@polymat.eu (M.S.); 3 IKERBASQUE, Basque Foundation for Science, 48009 Bilbao, Spain

**Keywords:** PBS-*ran*-PCL, activation energy of flow, microstructure, rheology, tackiness, HMA

## Abstract

The sole effect of the microstructure of biodegradable isodimorphic poly(butylene succinate)-*ran*-poly(ε-caprolactone) random copolyesters on their rheological properties is investigated. To avoid the effect of molecular weight and temperature, two rheological procedures are considered: the activation energy of flow, *E_a_*, and the phase angle versus complex modulus plots. An unexpected variation of both parameters with copolyester composition is observed, with respective maximum and minimum values for the 50/50 composition. This might be due to the peculiar chain configurations of the copolymers that vary as a function of comonomer distribution within the chains. The same chain configuration variations are responsible for the isodimorphic character of the copolymers in the crystalline state. Tack tests, performed to study the viability of the copolyesters as environmentally friendly hot melt adhesives (HMA), reveal a correlation with rheological results. Tackiness parameters, particularly the energy of adhesion obtained from stress-strain curves during debonding experiments, are enhanced as melt elasticity increases. Based on the carried-out analysis, the link microstructure-rheology-tackiness is established, allowing selecting the best performing HMA sample considering the polymer chemistry of the system.

## 1. Introduction

The worldwide production of all plastics was 348, 359, and 368 million metric tons in 2017, 2018 and 2019; however, the global plastics production fell by 0.3 percent in 2020 compared to the production in 2019, due to the COVID-19 pandemic [1]. Approximately 95–99% of polymer materials are produced from non-renewable sources (synthetic plastics) derived from petrochemicals. These products do not have a capacity for physical, chemical, and biological degradation and then contribute to increased environmental pollution [2,3].

Biodegradable polymers appeared as an alternative approach for many applications to control the hazard caused by synthetic (non-biodegradable) plastics. The production of biodegradable polymers requires 65% less energy than synthetic polymers [4,5]. Currently, biopolymers represent around 2.11 million tons produced in 2019 [1]. However, the market for biopolymers is continuously rising and diversifying.

Nowadays, biopolymers are used in different industrial sectors like rigid and flexible packaging due to their sustainability and reduction in environmental impact associated with the disposal of synthetic polymers. The most important application of biodegradable polymers in the packaging sector is their use as hot melt adhesives (HMAs). HMAs are polymer-based solvent-free thermal solid materials at room temperature that are applied in the molten state and consolidate adhesion with moderate to high strength bonds upon cooling [6,7]. The applications of these HMAs are in electronics (encapsulation and wire tacking), automobiles (interior trimming and seating assembly), packaging (pallet stabilization, box, and carton sealing, wrapping and case closings, appliances (gasket installation and trim), and furniture (assembly of panels and cabinets).

Usually, the base polymer of a HMA is a polyolefin like ethylene-vinyl acetate (EVA) copolymers or polyethylene (PE). Still, other polymers, such as polyamides, polyesters, polycarbonates, polyurethanes, polypyrrole, and polycaprolactone (PCL) are also used [8,9,10,11,12,13,14,15,16,17,18]. Specifically, PCL is an aliphatic semi-crystalline, biodegradable, easy to process, and cheap fossil-based polyester that can be employed for packaging.

On the other hand, polybutylene succinate (PBS) is a semi-crystalline thermoplastic aliphatic copolyester, biodegradable, with good processability, thermal stability, chemical resistance, and mechanical properties similar to those of PE and polypropylene (PP). Using both PCL and PBS as substitutes for non-biodegradable synthetic polymers is environmentally beneficial. Considering this, the combination of caprolactone and butyl succinate monomers to obtain random or block copolymers in the field of new materials has been contemplated in literature. In particular, our group has recently investigated the synthesis, thermal properties, and morphology of random PBS-*ran*-PCL copolyesters [19,20,21,22,23,24].

Within this framework, the rheological features and tackiness of random PBS-*ran*-PCL copolyesters, covering a wide range of compositions, have been investigated. Indeed, a sound analysis of the rheological parameters of random copolymers requires suppressing the influence of temperature and molecular weight, to focus exclusively on the effect of the proportion of monomers, i.e., the microstructure of the copolymer. This is often forgotten in the vast number of papers published on the rheology of copolymers in general. Typical rheological functions, such as Newtonian viscosity *η*_0_ and steady-state shear compliance, $J_{e}^{0}$, are both affected by molecular weight and temperature. Obtaining copolymers of different compositions and the same molecular weight is very difficult, and it is rarely considered. The case of random aromatic PES/PEES (polyethersulfone/polyetherethersulfone) copolymers of an estimated molecular weight of *M_n_* ≈ 9500 g/mole is unique in this sense [25]. The results show a depression of viscosity at intermediate compositions linked to an unexpected minimum of the glass transition temperature, which is allegedly due to an increase in free volume with the decrease in the number of ether groups in the chain.

In our work, two rheological procedures are proposed to avoid the undesirable effect of the aforementioned factors (molecular weight and temperature) and so obtain sound results, which account solely for the effect of the microstructure of the copolymers of different compositions: analysis of the activation energy of flow and analysis of plots of phase angle *δ* versus the absolute value of the complex modulus |G*|.

Progress of polymer rheology owes very much to the efforts of many researchers to link rheological measurements to polymer processing. Typical processing methods like extrusion, injection, and additive manufacturing have been soundly studied through rheological analysis. A relevant sustainability aspect of polymers, often ignored by environment researchers, is their low energy consumption during processing, as compared to other materials like glass, steel, or aluminum [26]. Needless to say, analyzing the flow in polymer processing at the industrial level requires non-linear regime rheological studies, limiting the reliability of the analyses in the linear regime. However, in the case of adhesives, advantage has been taken from linear viscoelastic results to study their applicability. In particular, for hot-melt adhesives, a low viscosity is required for a good extrusion in the gun and easy spreading, but a good immediate adhesion (called “tack”) to the surfaces to be welded is also necessary. The level of adhesion is measured through debonding tests (so-called probe-tack tests) in which the force to separate two welded surfaces is measured as a function of time, giving rise to a stress-strain curve.

In this paper, basic and applied characteristics of PBS-ran-PCL copolyesters are studied, focusing on the effect of microstructure on linear viscoelasticity and tack performance, for their use as HMA_s_. One important aim is to establish correlations between the melt elasticity of the samples and characteristics parameters of tack tests, such as tensile modulus in the linear zone, stress peak, stress plateau, and energy of adhesion.

## 2. Materials and Methods

### 2.1. Materials

**Scheme 1:**
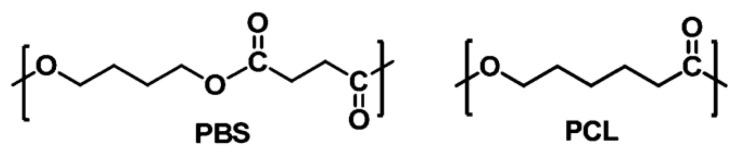
Chemical repeating units of PBS and PCL homopolyesters.

Four random copolymers *BS_x_CL_y_*, where *x* and *y* are the molar percentage of BS and CL unit respectively, and their parent homopolymers (PBS and PCL, Scheme 1) were analyzed in this work.

They were synthesized by a metal-catalyzed melt-polymerization in the presence of titanium tetraisopropoxide (TTP) as the catalyst, as described by Penas et al. [24]. Relevant data for these materials, corresponding to the high molecular weight (denoted as *HM_w_-BS_x_CL_y_*) reported by Penas et al. [24], are presented in Table 1.

**Figure 1:**
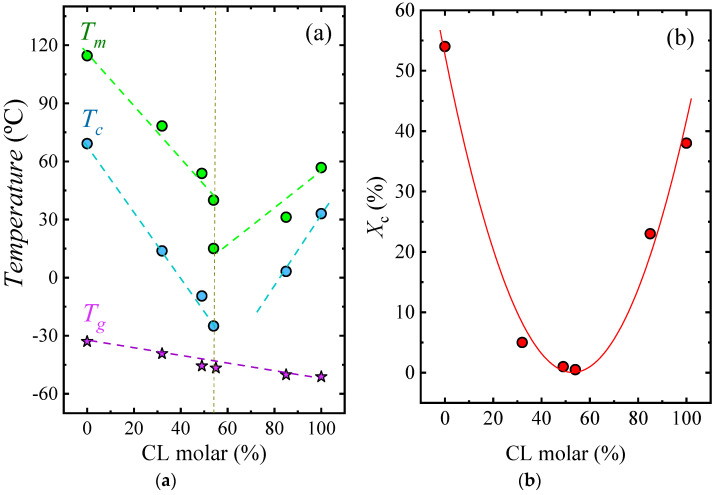
(**a**) Phase diagram of the *HM_w_-BS_x_CL_y_* copolymers based on non-isothermal crystallization. The short-dashed upright line specifies the pseudo-eutectic point. (**b**) Crystallization degree (*X_c_*) for PBS-*ran*-PCL copolyesters as a function of composition. These data, taken from [22], are included to facilitate the interpretation of rheological and tack results of Section 3.1 and Section 3.2, respectively.

The phase diagram of the samples as a function of composition is shown in Figure 1a. The first-order transitions, crystallization (*T_c_*), and melting (*T_m_*) are seen to display a minimum with composition. This minimum is the so-called pseudo-eutectic point that is one of the characteristics of isodimorphic random copolymers [27]. Isodimorphic PBS-*ran*-PCL copolymers develop two crystallographic phases, one PBS-rich crystal phase to the left of the pseudo-eutectic point in Figure 1a, and one PCL-rich crystal phase to the right of the pseudoeutectic point. Therefore, in PBS-rich phases, only the PBS chain segments within the random copolymer crystallize in unit cells that are very similar to those in neat PBS but contain a minority of PCL comonomer inclusions. Conversely, in PCL-rich phases, the PCL segments can pack in PCL like unit cells but contain small amounts of PBS comonomeric inclusions. We have previously demonstrated using Wide Angle X-ray Scattering (WAXS) that the crystalline unit cell dimensions change as a function of comonomer inclusion [20,21]. We have also found that at the pseudo-eutectic point, both PBS-rich and PCL-rich crystals can be formed. This is the reason why there are two melting points reported in Figure 1a for the composition at the pseudo-eutectic (signaled by a vertical line).

Previous works from our group [20,21,24] have demonstrated that PBS-*ran*-PCL copolymers are isodimorphic (i.e., can form two crystalline phases depending on composition as explained above) because: (a) they crystallize in the full composition range, despite being random, (b) they display a pseudo-eutectic point and (c) their crystal unit cell parameter are a function of composition.

This background is very important to realize that the copolymer chain microstructure changes with composition as the content of PBS (or PCL) increases in the copolymer. The crystallinity degree also follows a trend similar to the *T_m_* values versus composition (see Figure 1b). It decreases as PCL content increases and goes through a minimum at the pseudo-eutectic point only to increase again for PCL-rich compositions.

The chemical constitution or configuration of the copolymer chain starts with only PBS comonomeric units (when PBS homopolymer is considered as a starting point) that can perfectly pack to form PBS crystals. As PCL comonomeric units are incorporated randomly, the configuration of the chains changes, in such a way, that there is a competition between chain inclusion (favoring crystallization) and chain exclusion (favoring the exclusion of comonomeric units to the amorphous part and hence decreasing the crystallinity degree). The crystallinity degree of the PBS-rich compositions decreases until it reaches a minimum at the pseudo-eutectic behavior (Figure 1b). At higher PCL contents, the copolymer chain configuration has changed so much that PBS crystals are no longer formed, and only the PCL comonomeric units can crystallize (with some PBS comonomeric inclusions).

The amorphous part of the copolymers is miscible, as comonomeric units are covalently bonded. As a result, only one glass transition temperature is observed that depends on copolymer composition as shown in Figure 1a.

### 2.2. Rheological and Tack Experiments

Rheological and tackiness properties of the materials considered here were determined using an ARES strain-controlled rheometer (TA instrument). Parallel plates with diameters of 25 mm and 8 mm, were used respectively. For comparison purposes, the commercial hot melt adhesive Valco-PM 357 (Valco Melton SLU (Navarra, Spain)) was used.

#### 2.2.1. Rheology

A gap thickness of about 1 mm was used during rheological characterization of the materials considered here. Before frequency sweeps, all samples were heated and kept for 10 min at 40 °C above their melting temperatures (*T_m_*), inside the rheometer, to avoid the presence of any possible self-nuclei in the melt (i.e., to erase the crystalline memory of the material) [28].

Frequency sweeps were performed from 0.1 to 100 rad/s with a deformation level of 5%, which was within the linear viscoelastic regime as previously determined from strain sweeps. Four temperatures were applied between *T_m_* and *T_m_* + 40 (always starting from *T_m_ +* 40 °C and then decreasing the temperature to avoid any memory effects). A master curve of each material was determined from time-temperature superposition data using the TA Orchestrator V7.2.04 (TA Instruments, New Castle, DE, USA) software.

The Newtonian viscosity, *η*_0_, was determined from each frequency sweep by means of Equation (1). Its numerical value was determined from the Briedis and Faitelson model [29] using Equation (2). The effect of temperature on viscosity, was assessed through the Arrhenius Equation (3):

(1) $$\eta_{0}=\underset{\omega \to 0}{\lim}\eta^{\prime }$$

(2) $$\eta^{\prime }=\frac{\eta_{0}}{1+{\left(k\;\omega \right)}^{n}}$$

(3) $$\eta_{0}\left(T\right)=Be^{\left(E_{a}/RT\right)}$$

where *η*′ is the real part of the complex viscosity η*, *η*′ = *G*″/*ω*, *k* is a characteristic time, *n* is the slope of the linear zone of *η*′ at intermediate frequencies, *B* a pre-exponential factor which stands for the viscosity at a reference temperature, *R* the gas constant and *E_a_* the flow activation energy.

#### 2.2.2. Tack Experiments

**Figure 2:**
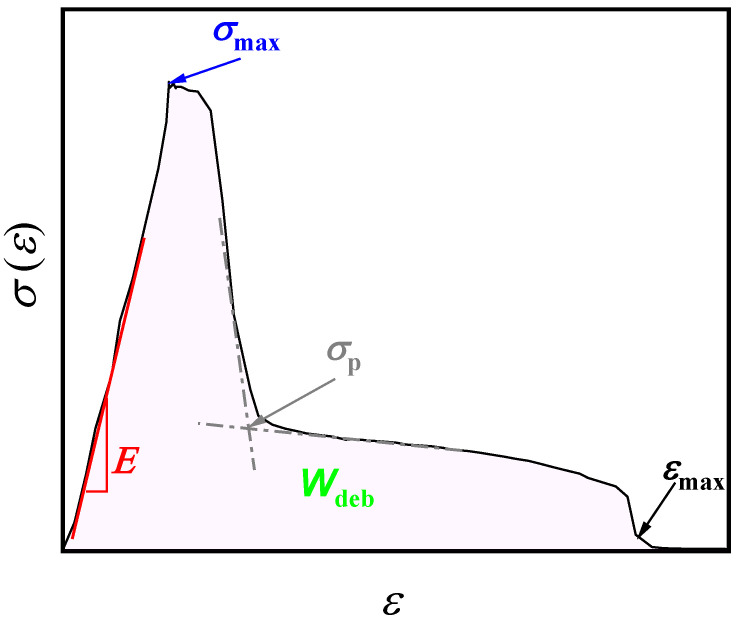
Schematic stress–strain curve obtained during probe-tack experiments illustrating the characteristic parameters extracted from the test (see text).

Tack experiments were carried out using an aluminum tack probe; the same kind of tests can also be performed using another material (e.g., a plastic or fiber sheet), which denotes the versatility of the procedure. In each case an amount of approximately 25 mg of unmolten material was placed in the lower plate so that, once molten, a material layer of 450 μm was formed. After ten minutes at *T_m_ +* 40, sample temperature was lowered to ten degrees above its melting temperature (*T_m_* + 10), and the probe-tack test was performed in three stages: (1) compression in which the upper plate descended at 0.1 mm/s until a material layer of *h*_0_ = 50 μm was formed, (2) contact period for 60 s, and (3) debonding at three different rates (*V_deb_*): 0.0314, 0.314 and 3.14 mm/s. At least two experiments were done at each debonding velocity. Force (*F*)–displacement (*h*(*t*)) data were acquired during each experiment and transformed to stress (*σ*)–strain (*ε*) curves (Equations (4)–(6)):

(4) $$\sigma =\frac{F}{A_{max}}$$

(5) $$\varepsilon =\left(h\left(t\right)-h_{0}\right)/h_{0}$$

(6) $$d\varepsilon /dt=\dot{\varepsilon }=V_{deb}/h_{0}$$

where *A_max_* is the contact area between the probe and the material during the compression stage, and *h*_0_ is the initial layer thickness. Plots of stress against the respective strain during debonding experiments yield typical curves, such as that presented in Figure 2.

From these curves, the following characteristic parameters can be obtained: (a) Tensile modulus *E*, in the linear regime (low strains or short times) (b) Stress peak, *σ_m_*_ax_, (c) Plateau stress *σ_p_*, (d) Maximum extension *ε_max_*, and (e) Work of debonding or adhesion *W_deb_* (i.e., the area under the loading curve multiplied by the initial layer thickness *h*_0_), as indicated by Equation (7):

(7) $$W_{deb}=h_{0}\int_{0}^{\varepsilon_{max}}\sigma \left(\varepsilon \right)d\varepsilon$$

## 3. Results

### 3.1. Small Amplitude Oscillatory Shear Measurements

**Figure 3:**
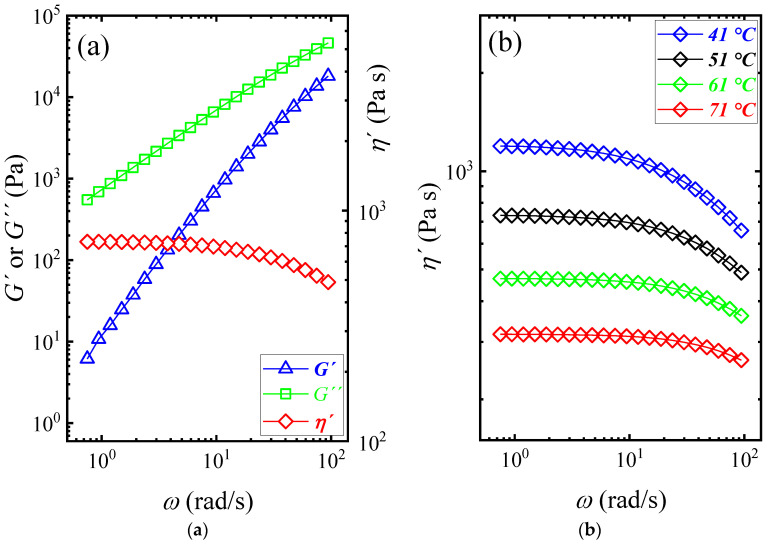
(**a**) Elastic and viscous moduli as a function of frequency for representative BS_15_CL_85_ copolyester at 51 °C. (**b**) Real part of the complex viscosity as a function of frequency for BS_15_CL_85_ copolyester obtained in a temperature range of 41–71 °C.

Frequency sweeps were obtained for all materials tested in a temperature range from *T_m_* to *T_m_* + 10 °C, at strain amplitudes corresponding to the linear viscoleastic regime. The study of large amplitude oscillatory shear tests, LAOS, in the nonlinear regime is out of the scope of the present work. Figure 3a shows elastic modulus, *G*′, and viscous modulus, *G*″, data for *BS_15_CL_85_* random copolymer at a temperature of 51 °C. The rest of the materials showed similar behavior. The results indicate that samples were essentially viscous in the frequency range studied (*G*″ > *G*′), which corresponds to the terminal region of the viscoelastic spectrum of polymers, as indicated by the slope values of viscoelastic moduli at low frequencies (i.e., close to 2 for *G*′ and to 1 for *G*″). In Figure 3b the real part of the complex viscosity, *η*′, is plotted as a function of frequency at different temperatures, also for *BS_15_CL_85_*. The Newtonian viscosity values *η*_0_ were obtained (Equation (2)) from these plots to evaluate the effect of temperature and determine the activation energy of flow, *E_a_* (Equation (3)).

As remarked in the Introduction, so far, it can be said that molecular weight is a hidden feature which alters and confounds the rheological results, especially viscosity data. On the other hand, it is in many cases assumed that the effect of temperature could be discarded taking rheological data at the same temperature for all compositions, omitting that the effect of temperature on viscosity depends on the distance to the glass transition temperature, *T_g_*. This error may be corrected by performing measurements at temperatures established at a value above *T_g_*, i.e., *T_g_ + k*, which indeed varies with the composition. In general, the inverse additive rule derived from the addition of the respective free volumes of the homopolymers [30,31] holds for the variation of *T_g_* with composition; this is observed, for instance, in the PBS-*ran*-PCL copolymers considered in this work, see Figure 1a (for more information see, [24]).

A rheological parameter that can be used to avoid the aforementioned effect of molecular weight and temperature in the rheology of random copolymers is the activation energy of flow, *E_a_*, which is obtained, among other methods, as explained in the Experimental part. In the range of temperatures suitable for polymer processing (estimated above approximately *T_g_* + 100), *E_a_* is independent of temperature. On the other hand, it is well known that the activation energy of flow becomes independent of the molecular weight above values corresponding to only 50–100 repeating units [29]. Indeed, a very interesting conclusion derived from the analysis of the effect of temperature on viscosity is that the activation energy of flow is only related to the chemical structure of the repeating unit. Some correlations have been found in literature, in particular between *E_a_* and the volume of the polymer pendant groups (e.g., polystyrene *E_a_* = 100 kJ/mol and polyethylene *E_a_* = 25 kJ/mol) and between *E_a_* and the polymer steric factor [32]. Therefore, it is assumed that the activation energy of flow increases with the rigidity of the polymer chain, which in turn depends exclusively on the chemical nature (microstructure) of the monomer [29], similarly to the glass transition temperature.

The second procedure to avoid the effect of temperature and molecular weight on the rheology of random copolymers, responds to the so-called Mavridis-Shroff-Van Gurp-Palmen plots [33,34,35,36,37], that is to say, phase angle *δ* versus the absolute value of complex modulus G*, |G*|, data, obtained at different frequencies and temperatures. For linear polymers and one-phase polymer blends and copolymers (like random copolymers), these systems give rise to one curve (master curve), which gathers all the compiled data denoting a thermo-rheologically simple behavior. On the contrary, typically, branched polymers, phase-separated polymer blends, and block copolymers bring about a thermos-rheological complex behavior, characterized not by a master curve but by different curves corresponding respectively to each temperature.

Notwithstanding the fact these rheological plots are sensitive to molecular weight poydispersity, it has been observed that at least for molecular weights above the entanglements molecular weight, *M_e_*, the master curve is independent of the average (weight or number) molecular weight: For each thermos-rheologically simple polymer sample, only one *δ* versus |G*| curve is obtained, which is independent of the temperature and the average molecular weight. For this reason, the use of Mavridis- Shroff-Van Gurp-Palmen plots allows one to determine two viscoelastic parameters, the phase angle *δ*, and the absolute value of the complex modulus |G*|, which depend only on the microstructure of the copolymer subsequent to the corresponding monomers proportion.

**Figure 4:**
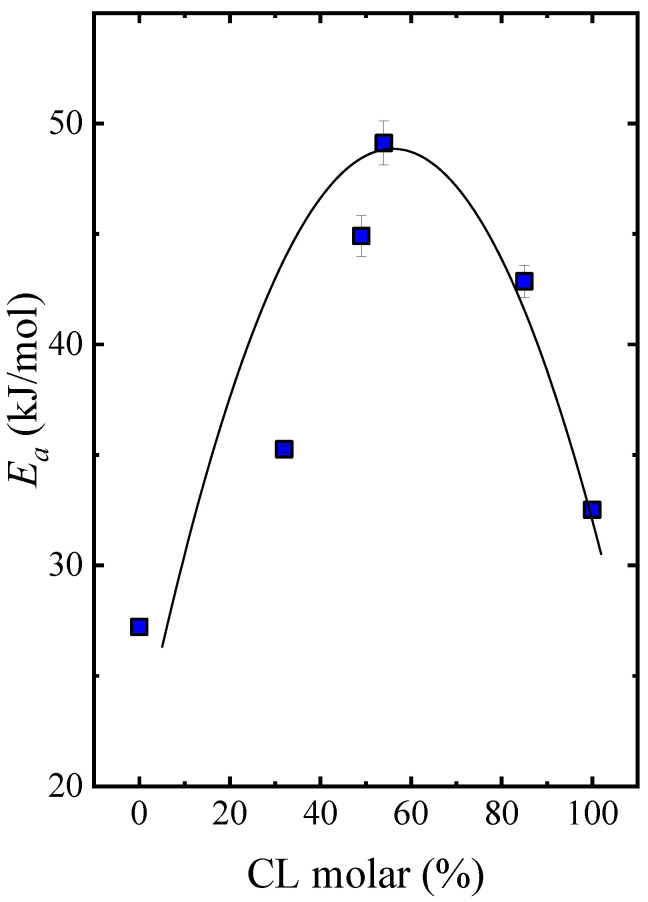
Activation energy of flow for PBS-*ran*-PCL copolyesters as a function of copolyester composition.

The temperature-independent activation energy of flow, *E_a_*, determined by means of Figures such as Figure 3b and Equation (3), is plotted as a function of comonomer composition for our PBS-*ran*-PCL copolyesters in Figure 4. The published studies on the effect of comonomer content on the activation energy of flow are just focused on the particular case of ethene/α-olefin copolymers, in which the effect of short-chain branching and long-chain branching is noticeable. A linear increase of *E_a_* with short-chain branching content (associated to α-olefin proportion) is reported by Stadler et al. [38], whereas Vega et al. [39] remark a deviation from this trend when long chain branches are present.

No results have been published in the literature about the sheer effect of comonomeric composition on the activation energy of flow of linear random copolymers (in the absence of branching), so our results do not have any possible comparison. Surprisingly enough, the data deviate from either a simple additive rule or an inverse additive rule showing, instead, a significant maximum for *E_a_* at a composition of PBS_46_/PCL_54_. Therefore, a linear increase of the activation energy of flow with comonomer content is not certainly observed in our case, which reasonably assumes that the arrangement of the comonomers at intermediate compositions would result in a rigidification of the copolymer chain. But this hypothesis is inconsistent with the glass transition temperature results, which are well fitted to the Gordon-Taylor equation (see Figure 1a), representing an inverse additive rule.

In an attempt to deepen in this rather striking result, we recall a work of Wang et al. [40] that remarks an unexpected *E_a_* maximum in a series of poly (1-olefins). Actually, the activation energy of flow passes through a maximum for poly (hexene-1) and poly (octene-1) and decreases for poly (hexadecane-1) and poly (octadecene-1), although the rigidity of the chains increases monotonically with the volume of side groups. Privalko and Lipatov [41] proposed an explanation of this anomaly, assuming that polymer melts would consist of regions with different instantaneous densities of packing, which leads to defining a “packing coefficient in the crystalline state”, *p_cc_*:

(8) $$p_{cc}=\left(\upsilon_{a}-\upsilon_{c}\right)/\upsilon_{a}$$

where *υ_a_* and *υ_c_* are, respectively, the specific volumes in the amorphous and crystalline states.

Privalko and Lipatov show (Figure 2 of [41]) that for a significant series of polymers, the lower the polymer packing coefficient in the crystalline state, the higher *E_a_* will be. Unfortunately, the works on the activation energy of flow of series of polymers with controlled microstructure have not found a continuity in contemporary times, which gives uncertainty to the hypothesis of packing. Still, our result of Figure 4 leads us to recall the crystallinity data of our PBS-*ran*-PCL random copolymers (see Figure 1b). As can be observed in this figure, the crystallinity degree of each copolymer plotted as a function of copolymer composition, shows a minimum at exactly the pseudoeutectic composition, i.e., PBS_46_/PCL_54_. As previously discussed above (in the experimental section), this minimum in crystallinity in isodimorphic random copolymers is a result of the exclusion/inclusion balance of comonomers in the crystalline regions, which in turn comes from the changes experienced in chain configuration as composition changes. The minimum in crystallinity, and the maximum in activation energy of flow, at intermediate compositions (both occurring at the same PBS/PCL ratio), might be related to a minimum in the polymer packing coefficient in the crystalline state, following the postulate of Privalko and Lipatov [41]. We can speculate that the changes in chain configuration, which originate the changes in crystallinity degree, also create changes in chain conformation and packing in the liquid state, leading to the peculiar behavior of the activation energy as a function of chain composition.

**Figure 5:**
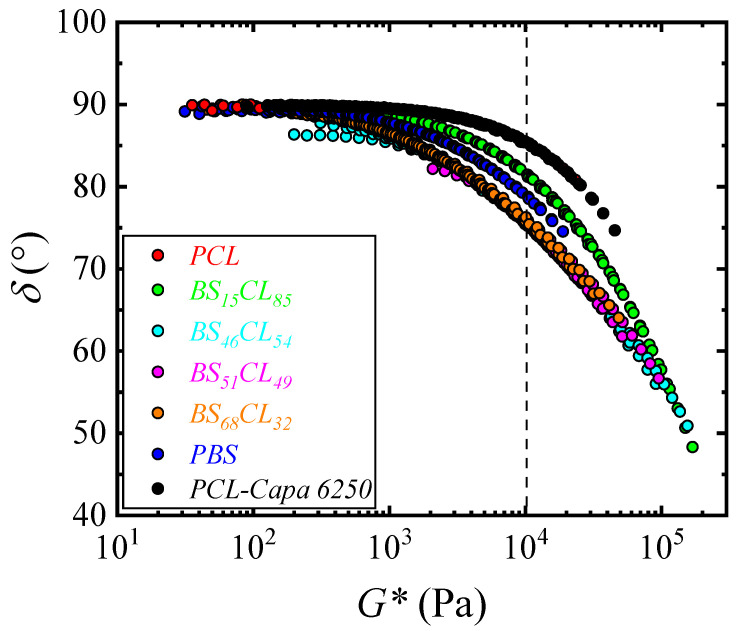
Mavridis-Schrof-Van Gurp-Palmen plots for the synthesized PBS-*ran*-PCL copolymers and commercial PCL Capa-6250. The vertical dash line at |G*|= 10^4^ Pa is drawn for the purpose described in the text.

Plots of phase angle *δ* as a function of the absolute value of the complex modulus, |G*|, obtained at different frequencies and temperatures (the so-called Mavridis-Schroff- Van Gurp-Palmen plots) for the different PBS-*ran*-PCL copolymer compositions are shown in Figure 5. These types of rheological plots have been reported for random copolymers, in particular for a series of ethylene/butene copolymers in a study centered on the effect of short-chain branching generated by butene comonomer [42].

In our case, each sample displays a different master curve which, in the absence of chain branches, stands for the sole effect of composition. According to literature results [36,37,43,44], the influence of the molecular weight is not reflected in *δ* versus |G*| plots, except at molecular weights below the molecular weight for entanglements, *M_e_*. This independence with respect to molecular weight is confirmed for PCL in Figure 5, where besides the sample considered in this work, data of another PCL with a different molecular weight (PCL-Capa-6250 M_w_ ≈ 25,000 g/mol) are presented: all data of both homopolymers lie on the same master curve.

**Figure 6:**
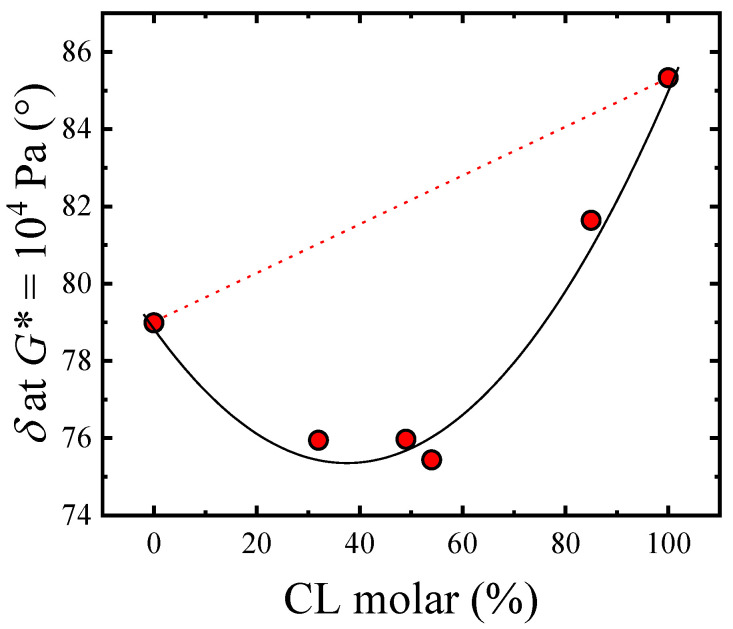
Phase angle *δ* read from Figure 5 as a function of PBS-*ran*-PCL copolymer composition. The dotted line represents the linear variation of *δ* with composition (additive rule). The solid line is a guide to the eye. The respective molecular weight polydispersity indexes of the samples are: P.I. (PBS) = 2.2, P.I. (BS_68_CL_32_) = 2.7, P.I. (BS_51_CL_49_) = 3.8, P.I. (BS_46_CL_54_) = 2.3, P.I. (BS_15_CL_85_) = 2.0, P.I. (PCL) = 1.9.

In an attempt to quantify the effect of copolymer composition on these viscoelastic results, we analyze the variation of the phase angle *δ* (which is a parameter reflecting the elasticity) taken at a constant complex modulus |G*| = 10^4^ Pa (vertical dash line in Figure 5) as a function of copolymer composition (Figure 6). The effect of composition is less noticed at lower constant complex modulus |G*|, because as frequency decreases, the elastic term is reduced, and viscosity tends to be the only component (*δ* approaches 90°).

The results, displayed in Figure 6, show a negative deviation from the additivity rule (dotted line) with a minimum at the pseudo-eutectic composition of (i.e., 46% PCL). However, an eventual effect of the polydispersity of the molecular weight distribution cannot be initially discarded since it has been probed in the literature that increasing the polydispersity index (P.I. = M_w_/M_n_) leads to a decrease of the phase angle *δ* for isotactic polypropylenes [45]. To analyze this hypothesis, the polydispersity indexes of our samples (presented in Table 1) are included in the legend of Figure 6: no dependency of the phase angle on the polydispersity is observed.

We note that the two investigated parameters, activation energy of flow (Figure 4) and phase angle *δ* are concomitant, with *E_a_* showing a maximum and a *δ* minimum at the same intermediate composition. But we should be cautious about the hypothesis of this coincidence being based on a minimum in the polymer packing coefficient in the crystalline state. On the contrary to the case of *E_a_*, no studies about the effect of microstructure on the melt elasticity in the linear viscoelastic regime (in terms of the elastic modulus, *G*′, or phase angle *δ*) have been published. Indeed, there is abundant literature on the effect of molecular weight, polydispersity, and long-chain branching of homopolymers [33,34,35,39,40,46,47,48,49,50], as well on the effect of interfaces in block copolymers and immiscible blends [51,52,53,54,55,56,57,58], but the literature about the effect of the chemical structure of the monomer on melt elasticity of polymers is inexistent. As stated above, it is known that broadening the molecular weight distribution leads to an increase of the melt elasticity (phase angle *δ* reduction), but this cannot be the reason of the *δ* minimum because combining the data of Table 1 and the results of Figure 6 no correlation between the corresponding values of P.I. and *δ* is observed

### 3.2. Implications of Linear Viscoelastic Results on Immediate Adhesion or Tack

The analysis of the correlation between immediate adhesion and viscoelasticity performed in last decades leads to conclude, in short, that predominantly elastic materials (with a phase angle *δ* < 45°) do not give rise to practically any debonding force, whereas viscous like materials (with a phase angle *δ* close to 90°) are characterized by a force which goes rapidly to a maximum and then decreases to zero, giving a low adhesion energy [59,60]. In the case of a balanced viscoelastic behavior, typically with a phase angle 45° < *δ* < 90°, a good tack is expected (high energy of adhesion) as pictured in the schematic graph shown in Figure 2, which represents the stress-strain curve during debonding experiment.

The frequently used Dahlquist criterion [61] is, in fact, a rule of thumb, which correlates the viscoelastic behavior of a hot melt adhesive or a pressure-sensitive adhesive to its tackiness. This criterion proposes that the tensile compliance *D*(*t*) should be 10^6^ Pa^−1^ or larger on the time scale of the bonding step (typically 1 s) to allow the creation of a contact surface with the solid surface. Thus, this is a sine qua non condition or a first step for tackiness. Actually, this value of the tensile compliance is equivalent to a shear compliance of *J*(*t*) = 3 × 10^−6^ Pa^−1^. Compliance is a viscoelastic function that stands for viscous and elastic response, obtained in the time domain by creep experiments.

Considering the correlation of time domain experiments to frequency domain experiments (e.g., Small Amplitude Oscillatory Shear or SAOS tests), the complex modulus may be expressed as G*(ω) = 1/J(t). Therefore, the Dahlquist criterion or the first condition to obtain tack, can be expressed as |G*| < 3.3 × 10^5^ Pa at a frequency of 1 rad/s. In literature, the criterion frequently used is *G*′ < 3.3 × 10^5^ Pa at a frequency of 1 rad/s, which is only correct if *G*″ ≫ *G*′, recalling that the absolute value of complex modulus is |G*| = [(*G*′)^2^ + (*G*″)^2^ ]^1/2^. This rule has been amplified by the less diffused Chang criterion [62]; accordingly, good tack is obtained if the elastic modulus lies between *G*′ = 3.3 × 10^5^ and *G*′ = 10^4^ Pa in a frequency range of 10^−2^ rad/s to 10^2^ rad/s.

As a first approach to the adequacy of our viscoelastic results to a tacky response, the Mavridis-Shroff-Van Gurp-Palmen plots (*δ* versus |G*| obtained in a frequency range of 10^−1^ rad/s to 10^2^ rad/s) in Figure 5, show that the Dahlquist criterion, |G*| < 3.3 × 10^5^ Pa, is fulfilled by our samples. Notwithstanding the industrial echo of the Dahlquist criterion, we have to assume that the process of adhesion joint failure under a debonding force, shown in Figure 2, is rarely matched with viscoelastic parameters [63]. In fact, as remarked by Lindner et al. [64] the debonding mechanism implies cavitation, fibrillation, elongation of fibrils, and strain hardening behavior at large strains, which exceed the framework of linear viscoelastic experiments in shear mode.

**Figure 7:**
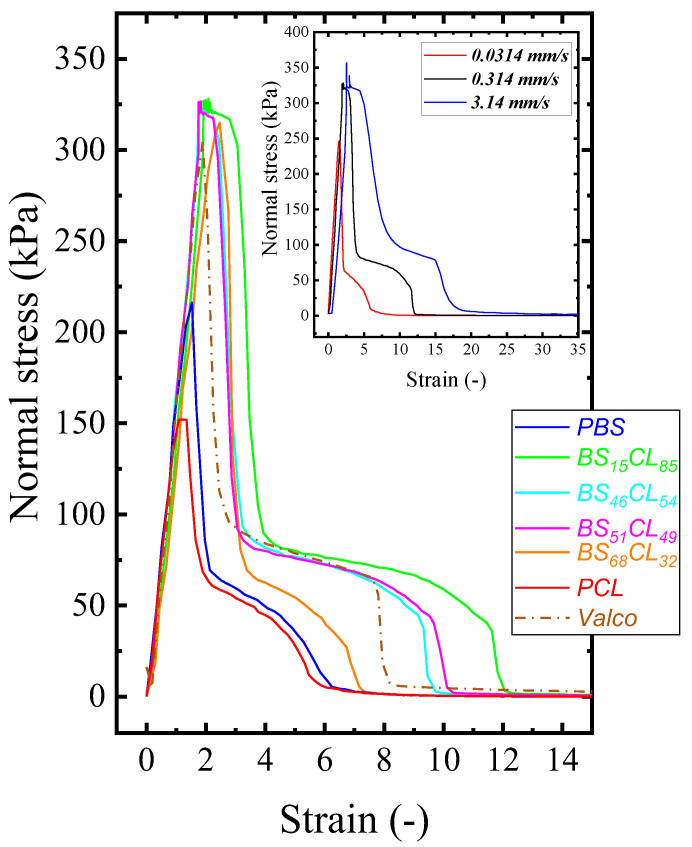
Stress-strain curves at different compositions obtained from probe-tack experiments and the same relative temperature with respect to *T_m_ (T_m_* + 10). The inset graph represents the effect of debonding velocity for BS_15_CL_85_ copolyester obtained a 41 °C. In all cases, fibrillation was observed.

Representative tack test results of our samples are shown in Figure 7, together with the results obtained with a commercial adhesive, plotted for comparison purposes. The experiments were performed at the corresponding *T_m_* + 10 °C temperature of each sample, because this is typically the application temperature for hot-melt adhesives. In the inlet of the Figure 7, results at different debonding velocities are shown for one of the samples, although the study of the effect of debonding velocity is not contemplated in this paper.

Microscopic studies of the adhesives carried out during debonding tests [59,64,65,66,67] show that reaching the maximum stress is related to the growing of cavities after cavitation starts, whereas fibrillation occurs at the stage that corresponds to the stress plateau. The corresponding values of the characteristic parameters of our samples and the commercial hot melt adhesive are presented in Table 2.

In spite of the aforementioned unclear perspectives to link debonding parameters to linear viscoelastic results, we have tried to establish and discuss experimental correlations between the most significant parameters of Figure 2 and Figure 7 (shown in Table 2) and the melt elasticity given by phase angle, *δ* (the lower *δ* the higher melt elasticity and vice versa).

**Figure 8:**
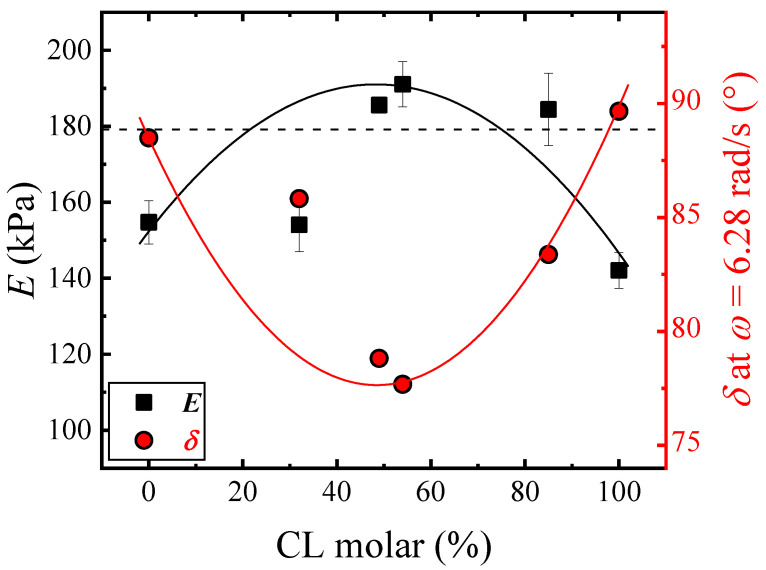
Tensile modulus *E* obtained at a debonding velocity of 0.314 mm/s (ω = 6.28 s^−1^) and phase angle *δ* obtained at ω = 6.28 rad/s, as a function of composition. The horizontal segmented line corresponds to the *E* value of the Valco adhesive. The solid lines are guides to the eye.

We first consider the elastic tensile modulus in the linear regime, E, obtained from the straight-line slope, observed at low strains in stress-strain curves of debonding tests (Figure 2). Since this is a parameter in the linear regime, it should be matched with a parameter that stands for the elasticity in SAOS experiments. Considering our results of Figure 5 and Figure 6, we take the phase angle *δ* as representative of melt elasticity, in terms of the expressed above. For correspondence between the results of debonding tests and SAOS results, the data of the latter should be taken at a frequency of *ω* = 6.28 rad/s, equivalent to a debonding velocity of 0.314 mm/s. The results shown in Figure 8 indicate an inverse trend for both parameters, with a maximum for the tensile modulus, *E*, and a minimum for phase angle *δ* at intermediate compositions, remarking that the more elastic the copolymer is (lower *δ*) the higher is the tensile modulus obtained in tack tests.

**Figure 9:**
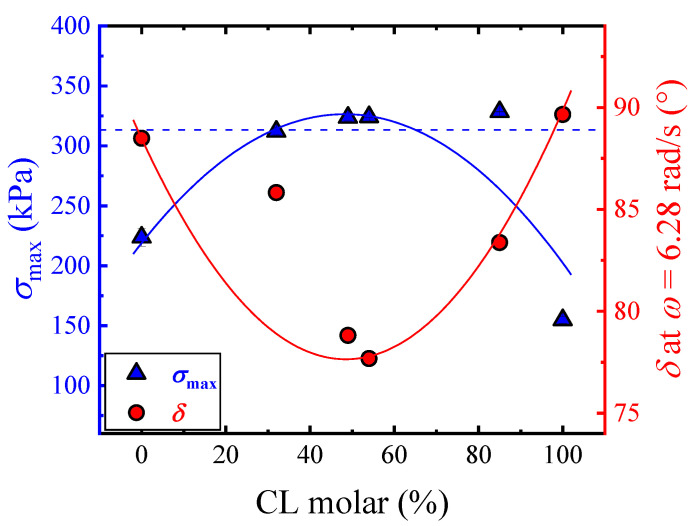
Stress at peak, *σ_max_*, obtained at a debonding velocity of 0.314 mm/s (*ω* = 6.28 s^−1^) and phase angle *δ* obtained at ω = 6.28 rad/s, as a function of composition. The horizontal segmented line corresponds to the *E* value of the Valco adhesive. The solid lines are guides to the eye.

Recalling the debonding test results of Lakrout et al. [59], Creton and Ciccoti [68] established that expansion or growth of cavities is related to the melt elasticity of the adhesives since it implies small local elastic deformations. Thus, the stress peak *σ_max_*, of tack tests, related to the growing of cavities, should increase with elastic modulus. This is observed by Lakrout et al. [59] when they analyze the effect of debonding velocity on stress-strain curves. In our case, the effect of the copolymer composition on stress peak at a constant debonding velocity (0.314 mm/s) has been analyzed. The practically inverse trend of *σ_max_,* and *δ* (inverse of melt elasticity) for our samples is shown in Figure 9. Stress peak *σ_max_*, values deviate positively from linearity (with a maximum), correlatively to a negative deviation of *δ* (with a minimum). Hence, the more elastic the copolymer, the higher the stress peak in stress-strain curves such as those of Figure 7. This result confirms the reported correlation between elasticity and *σ_max_* [59], but the complexity of the debonding process cannot be ignored. As pointed out by Chiche et al. [69] *σ_max_* depends also on the size and density of the initial defects present at the adhesion interface. In view of the clear synergic effect of melt elasticity on *σ_max_* observed in Figure 9 it can be assumed that, in our case, the process of creation of initial defects during the compressive contact stage is not significantly different for each composition.

**Figure 10:**
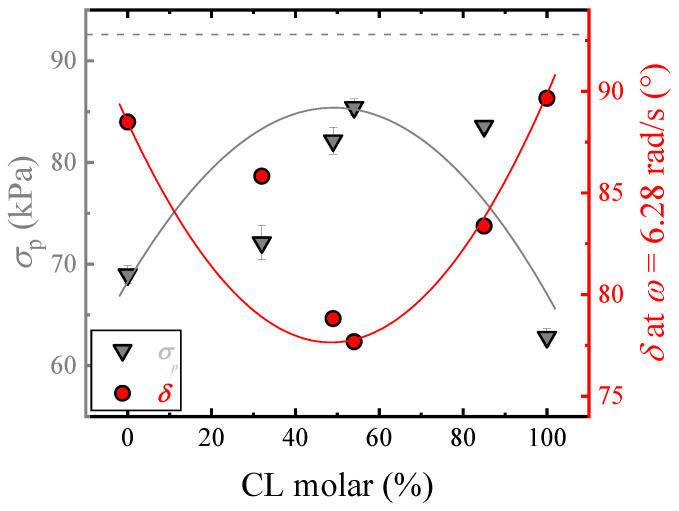
Stress at the plateau of stress-strain curve, *σ_p_* obtained at a debonding velocity of 0.314 mm/s (ω = 6.28 s^−1^) and phase angle *δ* obtained at ω = 6.28 rad/s, as a function of composition. The horizontal segmented line corresponds to the value of *σ_p_* for the Valco adhesive. The solid lines are guides to the eye.

According to Chiche et al. [69], the stress in the plateau of stress-strain curves, *σ_p_* (Figure 2 and Figure 7) can be more unequivocally associated only with the elasticity of the adhesive. Actually, *σ_p_* stands for the process of collective growth of the cavities as foam and it is independent of how the adhesive surface is formed at the beginning of tack tests. The effect of copolymer composition on both, stress plateau, *σ_p_*, and phase angle *δ* is shown in Figure 10: Increasing melt elasticity (lower *δ* values at intermediate compositions) leads to enhanced values of *σ_p_*.

**Figure 11:**
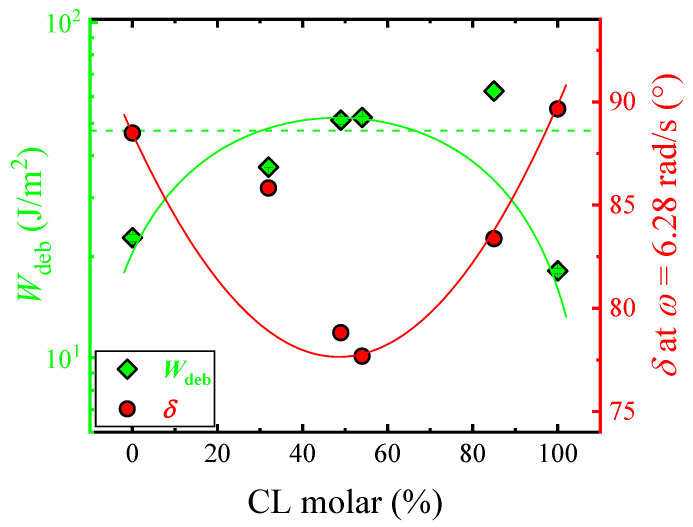
Energy of adhesion *W_deb_*, obtained at a debonding velocity of 0.314 mm/s (ω = 6.28 s^−1^) and phase angle *δ* obtained at ω = 6.28 rad/s, as a function of composition. The horizontal segmented line corresponds to the value of *W_deb_* for the hot melt Valco. The solid lines are guides to the eye.

The energy of adhesion or debonding energy, *W_deb_*, which is obtained from the area under the stress-strain curve, is determined by the growth of the cavities (linked to stress peak *σ_max_*) and subsequent fibrillation process. Therefore, it is expected to increase with the melt elasticity of the samples, expressed in terms of the phase angle, *δ.* The variation of *W_deb_* with the composition of the copolymer is shown in Figure 11, together with melt elasticity *δ* data, showing the same trend as the other parameters defined in stress-strain curves of tack tests.

Our results lead us to deduce that the effect of the microstructure of the PBS-*ran*-PCL copolymers on linear viscoelasticity, characterized by a maximum in the activation energy of flow and a minimum of the phase angle *δ* at intermediate compositions, is also reflected on the parameters of tack tests: tensile modulus in the linear zone of the strain-strain curve, stress peak, stress plateau and energy of adhesion are enhanced as melt elasticity increases, i.e., *δ* decreases. This affects the practical suitability of the investigated copolymers for their use as hot melt adhesives. Particularly, the summary of the results shown in Table 2 indicates that PBS-*ran*-PCL copolymers offer better tack than the respective PBS and PCL homopolymers. Moreover, our data compare well with those of a commercial Valco adhesive, with unequivocally adequate tack performance for daily use. Although overall, the _15_ PBS/_85_ PCL composition offers the best tack properties, its low melting temperature, *T_m_* = 31 °C, invalidates its use as a hot melt adhesive. An analysis of the thermal properties (Table 1) and tack performance (Table 2), leads to conclude that PBS_51_/PCL_49_ (*T_m_* = 53.8 °C) is the most balanced and appropriate for practical purposes.

## 4. Conclusions

Respective analysis of the activation energy of flow and plots of phase angle *δ* versus |G*|, proposed to investigate the linear viscoelasticity of random copolymers avoiding the effect of molecular weight and temperature, bring about rather surprising results, for the investigated PBS-*ran*-PCL random biocopolymers. At the current level of knowledge about these materials, we assume that the observed *E_a_* maximum at intermediate compositions might be due to a minimum in the polymer packing coefficient in the crystalline state. This correlates with the minimum observed in the crystallinity degree of the copolymers at the pseudo-eutectic composition. Such minimum is originated in the change in polymer chain configuration with composition leading to an inclusion/exclusion competition in the crystalline/amorphous states that depends on composition. Regarding the minimum of the phase angle *δ* at intermediate compositions, it constitutes a unique case of the exclusive effect of the microstructure of the copolymer on the melt elasticity, but we have not a clear explanation about it, so far. In any case, it is demonstrated that this minimum is not related to the polydispersity of the molecular weight distribution. With respect to the implications of SAOS results on the application of the studied biocopolymers to the field of adhesives and sealants, the following conclusions can be drawn:

The characteristic parameters of the stress-strain curves of tack probe tests, i.e., tensile modulus in the linear zone, stress peak, stress plateau, and energy of adhesion, increase with enhancing the melt elasticity of the copolymer.

The copolymers show a better tack performance than the respective homopolymers and require less energy consumption for the spreading process, because of their lower melting temperatures. From a practical point of view, the PBS_51_/PCL_49_ random copolymer is the best sample, showing tack properties (in particular energy of adhesion) as good as those observed for a commercial adhesive used for comparison purposes.

## Figures and Tables

**Table 1:** Composition, molecular weight, and thermal characteristics of the PBS-*ran*-PCL [24].

	Composition ^a^	Molecular Weight ^b^ (g/mol)			*T_g_* ^c^	*T_m_* ^d^
Polyester	(*BS/CL* mol/mol)	*M_w_*	*M_n_*	*P.I.*	(°C)	(°C)
PBS	100/0	70,300	32,700	2.2	−31.7	114.6
BS_68_CL_32_	68/32	91,300	34,000	2.7	−42.8	78.3
BS_51_CL_49_	51/49	87,850	23,200	3.8	−45.3	53.8
BS_46_CL_54_	46/54	60,700	26,000	2.3	−47.3	40 & 15
BS_15_CL_85_	15/85	53,100	26,350	2.0	−55.1	31.2
PCL	0/100	60,000	31,000	1.9	−60.1	56.8

^a^ Composition as determined by ^1^H-NMR. ^b^ Weight-and-number average molecular weights and polydispersity index. ^c^ Glass transition temperature. ^d^ DSC Melting temperature.

**Table 2:** Tack characteristic parameters obtained from the stress–strain curves at $\left(T_{m}+10\right)$ and a $V_{deb}$ of 0.314 mm/s.

		Characteristic Parameters			
Material	(*T_m_* + 10) °C	E	$\sigma_{max}\;(\mathrm{kPa})$	$\sigma_{p}\;(\mathrm{kPa})$	$W_{deb}\;(J/m^{2})$
PBS	125	154.7 ± 5.7	223.7 ± 7.6	69.0 ± 0.9	22.8 ± 0.6
BS_68_CL_32_	88	154.1 ± 7.1	312.1 ± 2.8	72.1 ± 1.7	37.0 ± 0.1
BS_51_CL_49_	64	185.6 ± 0.1	323.7 ± 2.9	82.1 ± 1.3	51.1 ± 1.4
BS_46_CL_54_	50	191.0 ± 5.9	324.2 ± 0.6	85.4 ± 0.8	52.0 ± 0.5
BS_15_CL_85_	41	184.4 ± 9.5	328.4 ± 0.2	83.6 ± 0.6	62.3 ± 3.6
PCL	67	142.1 ± 4.7	154.8 ± 2.8	62.8 ± 0.9	18.1 ± 0.3
Valco	70	179.2 ± 20.2	313.3 ± 9.0	92.6 ± 0.5	47.5 ± 3.0

## Data Availability

The data presented in this study are available on request from the corresponding author.
